# Factors associated with dietary behaviour in kidney transplant recipients: a cross-sectional study

**DOI:** 10.3389/fnut.2025.1467667

**Published:** 2025-06-10

**Authors:** Jiaxin Fang, Xiaohong Lin, Changyun Wei, Qianlu Wang, Keke Lin, Shuping Zhang, Peiyu Zhao, Xiangru Li, Hongxia Liu

**Affiliations:** ^1^School of Traditional Chinese Medicine, Beijing University of Chinese Medicine, Beijing, China; ^2^School of Nursing, Beijing University of Chinese Medicine, Beijing, China; ^3^Nursing Department, China-Japan Friendship Hospital, Beijing, China

**Keywords:** kidney transplant recipients, dietary behaviour, depression, health promotion model, path analysis

## Abstract

**Background:**

Dietary behaviour among kidney transplant recipients (KTRs) is a critical factor influencing long-term postoperative outcomes. However, research focusing on the dietary behaviour of KTRs remains limited. The aim of this study was to examine the dietary behaviour and explore the influencing factors of dietary behaviour in KTRs based on a health promotion model.

**Methods:**

This was a cross-sectional study. A total of 289 KTRs from four hospitals were recruited from October 2022 to March 2023 by convenience sampling method. Participants completed a survey consisting of a general information questionnaire, the Dietary Self-management Ability Scale, the Perceived Dietary Behaviour Benefit and Barrier Questionnaire, the Self-Rating Depression Scale, and the Adapted Health Eating Cognitive Beliefs Questionnaire. The survey was administered both online and on-site. Data were analysed with Spearman’s correlation analysis and path analysis.

**Results:**

Dietary behaviour was at moderate level, with an index score of 78%. Depression (*β* = −0.207, *p* < 0.001), perceived behaviour benefits (*β* = 0.220, *p* < 0.001), perceived behaviour barriers (*β* = −0.234, *p* < 0.001), attitude (*β* = 0.135, *p* = 0.026), and social support (*β* = 0.166, *p* = 0.001) directly predicted dietary behaviour, while depression, attitude, and social support exhibited indirect effect on dietary behaviour.

**Conclusion:**

Medical staff could help KTRs change their poor dietary attitude and negative mode, guide them to receive more social support and perceived benefits, reduce their perceived barriers. These positive shifts could ultimately facilitate the emergence of healthy dietary behaviour and enhancement of quality of life.

## 1 Introduction

Kidney transplantation, the earliest solid organ transplantation to be clinically developed, is characterised by the largest number of transplantation cases and a highly advanced technology (1). According to the World Health Organization (2), the global count of kidney transplants in 2021 reached a total of 92,532.

Dietary behaviour among kidney transplant recipients (KTRs) is a critical factor that significantly influences their health outcomes. The Kidney Disease Outcome Quality Initiative (KDOQI) Clinical Practice Guideline for Nutrition in Chronic Kidney Disease recommends that KTRs maintain a protein intake of 1.0–1.2 g/kg body weight per day and an energy intake of 25–35 kcal/kg body weight per day, while also considering multivitamin and vitamin D supplementation, and managing electrolyte levels based on individual needs (3). Additionally, increasing fruits and vegetables and following a Mediterranean diet are suggested to improve overall health outcomes (3). A healthy dietary behaviour among KTRs has been proven to lower the risk of metabolic and cardiovascular diseases (4), contribute to the restoration of renal function, and reduce overall mortality (5).

KDOQI of the National Kidney Foundation has released evidence-based recommendations suggesting that disease diagnosis, level of physical activity, age, gender, weight status, nutritional knowledge, beliefs, and depression should be taken into account when assessing a recipient’s dietary intake and energy requirements (3). Additionally, previous international clinical studies have verified the influence of coping resources, such as social support, on the dietary habits of patients with chronic illness (6). However, there is a scarcity of theoretically-based research exploring the interplay between these variables and their effects on the dietary behaviour of KTRs.

Health Promotion Model (HPM), initially formulated in 1987 by American nurse practitioner Pender and her colleagues, is thought to be a paradigm for explaining health-promoting behaviour (7). The HPM classifies the factors influencing health-promoting behaviour into three primary categories. (i) Individual characteristics and experiences. Personal traits (physical, psychological, and sociocultural) and previously related behaviour are included. (ii) Behaviour-specific cognitions and affect, such as perceived benefit and barrier, perceived self-efficacy, activity-related affect, interpersonal influences from families and caregivers and situational influences. (iii) Behavioural outcome, such as healthy dietary behaviour. Grounded in HPM model, there are interactions between different behaviour-specific perceptions and emotion, for example, activity-related affect (dietary attitude) influencing perceived self-efficacy, perceived self-efficacy influencing perceived barriers, and self-efficacy being considered as a significant predictor of changes in health behaviour (8).

Over 50% of studies have found significant associations between health behaviour and factors such as perceived benefit and barrier, self-efficacy, and social support (9). Previous studies have demonstrated the significance of cultivating health beliefs and fostering attitudinal shifts to facilitate behavioural modifications (10), and individuals with positive attitudes exhibiting a greater propensity to adhere to health behaviour (11). And dietary attitude of female college students were negatively associated with their levels of depression (12).

**Figure 1:**
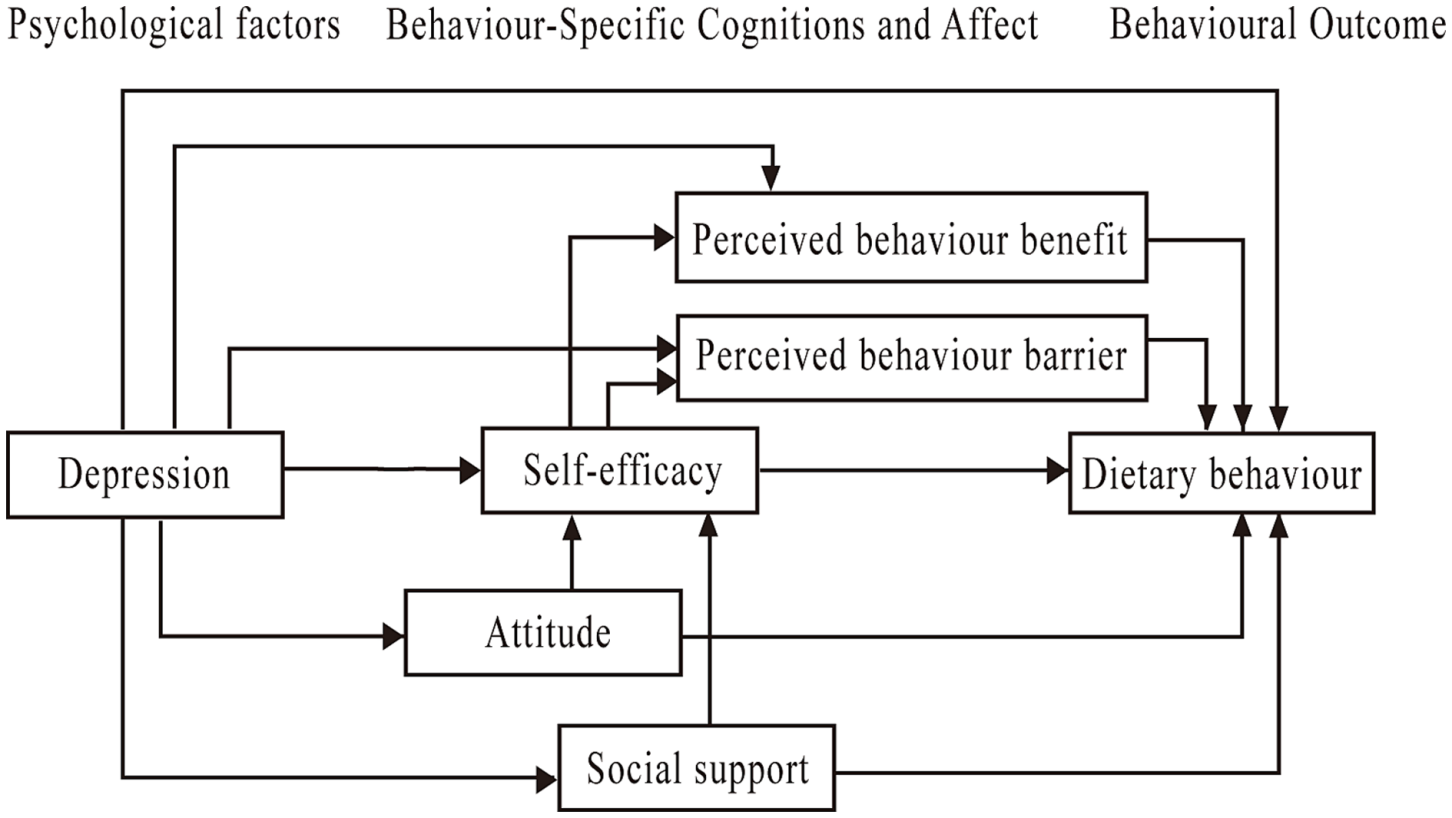
Initial hypothetical model based on HPM.

In addition, researchers have pointed out that there is a significant correlation between perceived self-efficacy and perceived benefit (13). Additionally, empirical evidence has demonstrated a positive correlation between social support and self-efficacy among patients with chronic diseases (14). Enhancing patients’ self-efficacy by means of providing social support has been found to have a positive impact on modifying health behaviours (15). Therefore, the purpose of our study was to employ the HPM for investigating the influencing factors of dietary behaviour in KTRs. The hypothetical theoretical model was formulated based on existing literature and theories (see Figure 1). Perceived benefit, perceived barrier, self-efficacy, depression, and attitude were considered to have a direct influence on dietary behaviour of KTRs. Depression could indirectly impact the dietary behaviour through perceived benefit, perceived barrier, self-efficacy, attitude, and social support. Self-efficacy was considered a common mediating variable between dietary attitude and perceived behaviour benefit, social support and perceived behaviour benefit, dietary attitude and perceived behaviour barrier, and social support and perceived behaviour barrier.

## 2 Materials and methods

### 2.1 Study design and participants

This was a cross-sectional study involving KTRs from four tertiary hospitals located in four provinces of mainland China. Participants were recruited between October 2022 and March 2023. Convenience sampling was used. Study design followed the Strengthening the Reporting of Observational Studies in Epidemiology (STROBE) statement (see Supplementary material).

Sample size calculation was determined based on five to ten times of the number of free parameters in the SEM (16). In this study, the number of free parameters in the initial model was 32. Considering a potential sample loss of 20%, a final sample size of 200–400 was necessary. Ultimately, a total of 343 questionnaires were distributed and 289 valid questionnaires were returned, with an effective recovery rate of 84.26%.

The inclusion criteria were as follows: (1) aged ≥18 years; (2) with a functional transplanted kidney; (3) with necessary reading and writing ability; and (4) voluntary participation. The exclusion criteria were as follows: (1) second or multiple kidney transplantation; (2) multiple organ transplant recipients; (3) with other severe diseases; and (4) pregnant women. KTRs who met the inclusion and exclusion criteria were invited to participate in the questionnaire survey.

### 2.2 Procedure

The study was approved by the Ethics Committee in Beijing University of Chinese Medicine (2023BZYLL0107). This study was conducted across multiple hospitals, and questionnaires were distributed and collected either on-site or online, depending on the circumstances at each hospital. KTRs were recruited from hospital transplant follow-up clinics. All participants received either verbal or written information about the study. Professionally trained researchers provided a detailed explanation of the study’s purpose and procedures, ensuring participants understood that withdrawing would not impact their future care. Following the completion of the informed consent forms, participants proceeded to fill out the questionnaires. All questionnaires used in this study were in Chinese. Additionally, all personal data concerning the individuals were anonymized.

### 2.3 Measures

A self-designed general information questionnaire, including demographic characteristics and disease-related information, was used to assess KTR’s general condition. Demographic characteristics included age, sex, BMI, ethnicity, habitual residence, employment status, education level, marital status, insurance status, and economic burden. Disease-related information included the time after transplantation, donor type, rejection, infection, and number of hospitalizations.

Dietary Self-Management Ability Scale was used to assess KTR’s dietary attitude and behaviours. The dietary attitude dimension contains 9 items with a score range of 9–45, investigating recipients’ willingness and levels of support for reasonable diet. For example, “I believe that reasonable diet after transplantation has a significant impact on physical and functional recovery.” The dietary behaviour dimension contains 10 items with a score range of 10–50, investigating dietary choice, intake and control, and physical activity. For example, “I adjust my daily water intake based on urine volume and body weight.” Each item was measured with a 5-point Likert scale from 1 (totally disagree) to 5 (totally agree). The scores for each dimension were calculated as the sum of the item scores within that dimension. Higher scores indicated better dietary attitude and behaviours. The scale has been validated in KTRs and has shown good psychometric properties (17). In this study, Cronbach’s *α* coefficient for the entire scale was 0.888.

Perceived Dietary Behaviour Benefit and Barrier Questionnaire was used to assess KTR’s perceived dietary behaviour benefits and barriers. The questionnaire was self-designed by the research team according to the research purpose, combined with a literature review and expert consultation. Each of the two dimensions contains 6 items. Each item was rated on a 5-point Likert scale from 1 (totally disagree) to 5 (totally agree). Higher scores indicated more perceived dietary behaviour benefits or greater perceived dietary behaviour barriers. For example, “it can help me maintain ideal body weight,” or “it is hard to follow the post-transplant diet when eating out frequently.” Seven experts were invited to assess item-level content validity (ICVI) and scale-level content validity (SCVI). Being equal or greater than 0.79 was considered relevant (18). In this study, expert SCVI was 0.929, and ICVI ranged from 0.857 to 1.000. Cronbach’s *α* coefficients of Perceived Dietary Behaviour Benefit and Barrier Questionnaire were 0.927 and 0.738, respectively.

Self-rating Depression Scale was used to measure KTR’s depression (19). It comprised 20 items and each item was rated on a 4-point Likert scale ranging from 1 (no) to 4 (always). Depression scores range from 20 to 80, with higher scores indicating more severe depression symptoms. The depression symptoms were evaluated based on standard scores. The total score was calculated by summing the scores of each item, and the result was then multiplied by 1.25. The standard score was obtained by rounding up to the nearest whole number. According to the Chinese norm results, the final standard scores were categorised as follows: normal (<50), mild (50–59), moderate (60–69), and severe (>70) (19). This scale has been validated in KTRs and has shown good psychometric properties (20). In this study, the Cronbach’s *α* coefficient of this scale was 0.860.

Adapted Health-Eating Cognitive Beliefs Questionnaire was used to assess KTR’s self-efficacy and social support. This questionnaire was adapted by the researchers based on Health Eating Cognitive Beliefs Questionnaire (21), according to literature reviews and expert consultations. The content evaluation of the questionnaire was assessed by experts. Modifications included changing the original scale from “healthy eating” to “healthy eating after transplantation,” and changing the 4-point Likert scale for the social support dimension to a 5-point Likert scale (1 = completely non-conforming, 5 = completely conforming). Self-efficacy scores range from 7 to 35, and social support scores range from 5 to 25, with higher scores indicating higher corresponding indicators. SCVI and ICVI values obtained from expert evaluations in this study were all 1.000, indicating good content validity. The Cronbach’s *α* coefficients of self-efficacy and social support were 0.811 and 0.812, respectively.

Scoring indicators of dietary behaviour and related factors in HPM were calculated using the following formula: the median score of each variable or dimension divided by the maximum possible score for that variable or dimension, multiplied by 100%. Based on these calculations, different variables were categorised into three levels: high (>80%), medium (60–80%), and low (<60%) (22).

### 2.4 Statistical analyses

Data were conducted employing SPSS 26.0 (IBM Corp.) and Mplus 8.3 (23). Two-sided tests were used with a significance level of *p* < 0.05. Descriptive data were presented as frequencies, percentages, means, standard deviations, medians and quartiles. Non-parametric test was adopted to ascertain demographic information and disease-related data pertaining to dietary behaviour. All statistically significant indicators were adjusted for multiple comparisons using the Bonferroni post-hoc test. The significance level after applying the Bonferroni correction for multiple comparisons was set to *α*′ = 0.05/n, where n represents the number of tests conducted. Spearman’s correlation was adopted to explore the association between dietary behaviour and various associated variables in HPM. The variance inflation factor (VIF) was employed to evaluate multicollinearity among the associated variables. VIF values of <5 indicate the absence of significant multicollinearity. VIF values between 5 and 10 suggest moderate multicollinearity, warranting cautious interpretation of the coefficients or consideration of merging/removing variables. VIF values of ≥10 indicate severe multicollinearity, necessitating remedial action.

To validate the hypothesized conceptual model, path analysis was used. Those variables that were significantly correlated with dietary behaviour entered into the path analysis to explore the influencing factors of dietary behaviour in KTRs. The collected data were incorporated into the path analysis for parameters estimation. The rationality of the parameters, the significance of the path, the goodness of fit, and other indices were integrated to assess the appropriateness of the sample data for the hypothetical model and the robustness of the model’s explanation power. If the hypothetical model exhibited a poor fit, it was necessary to correct the model. Through continuous evaluation, the most reasonable model with the best model fit and the strongest explanatory power was obtained. The fit of the sample data to the hypothetical theoretical model was assessed by various model indexes, including Chi-square degrees of freedom (χ^2^/df), Comparative Fit Index (CFI), Tacker-Lewis Index (TLI), Root Mean Square Error of Approximation (RMSEA) and Standardized Root Mean Square Residual (SRMR). Generally, χ^2^/df < 3, CFI and TLI > 0.90, RMSEA < 0.05, and SRMR < 0.08 indicate a good fit for the model, while RMSEA<0.08 are considered an acceptable fit (16, 24).

## 3 Results

### 3.1 Participant profile

Among the 289 KTRs, 58.82% were male. The mean age of the recipients was 44.37 ± 10.37 years. The mean Body Mass Index (BMI) was 22.20 ± 3.13 kg/m^2^, with the majority of recipients falling within the normal weight range. The majority of the recipients identified as Han ethnicity (93.43%). Most of the recipients resided in cities and towns (75.78%). A total of 51.56% of KTRs had attained college education or higher. The majority of recipients were married (79.24%), employed (52.94%), and relied on medicaid insurance payments (97.92%). Approximately 83.04% of KTRs experienced an economic burden. The time after transplantation ranged from 3 to 300 months. The percentage of deceased donors was 90.31%. In total, 20.42% of recipients had experienced rejection, and 28.03% experienced infection. Among the recipients, 67.13% were readmitted to the hospital.

The participants reported high levels of attitude and perceived behaviour benefits, as well as moderate levels of social support, self-efficacy, and perceived behaviour barriers. About 41.86% of participants were classified as depressed. Score of dietary behaviour was at a moderate level (Table 1).

**Table 1:** Scores of dietary behaviour and related factors (*n* = 289).

Variables	Median (P_25_, P_75_)/Mean ± SD	Range (points)	Index score (%)
Attitude	37.00 (35.00, 43.00)	21–45	82.22%
Perceived behaviour benefits	24.00 (24.00, 30.00)	16–30	80.00%
Perceived behaviour barriers	17.00 (14.00, 20.00)	6–29	56.57%
Self-efficacy	20.00 (17.00, 25.00)	9–35	57.14%
Social support	15.00 (13.00, 19.00)	5–25	60.00%
Depression	37.93 ± 8.06	20–61	–
Dietary behaviour	39.00 (34.50, 42.00)	15–50	78.00%

### 3.2 Univariate analyses of demographic and clinical characteristics

The results of the non-parametric test indicate a significant association between BMI and dietary behaviour among KTRs. Further analysis using the Bonferroni post-hoc test revealed a significant difference between the normal BMI group and the high BMI group (adjusted *p* = 0.001 < 0.017). Recipients without financial burdens exhibited better dietary behaviour (*p* = 0.011). With an increased post-transplantation duration, score of dietary behaviour decreased (*p* < 0.001). The Bonferroni post-hoc test indicated significant differences in score between recipients at 3–6 months and those at over 60 months post-transplant (adjusted *p* = 0.003 < 0.008), as well as between those at 13–60 months and those at over 60 months (adjusted *p* = 0.004 < 0.008). Recipients without a history of rejection demonstrated better dietary behaviour (*p* = 0.022). Detailed results are presented in Table 2.

**Table 2:** Univariate analyses of demographic and clinical characteristics (*n* = 289).

Variables	n (%)	Diet behaviour (points), Median (P_25_, P_75_)	Z/H statistics	*p*-value
Age				
18 ~ 45 years	150 (51.90%)	38.00 (33.75, 41.25)	1.665^b^	0.435
46 ~ 59 years	118 (40.83%)	39.00 (35.00, 42.00)		
≥60 years	21 (7.27%)	38.00 (36.00, 41.00)		
Sex				
Male	170 (58.82%)	39.00 (35.00, 42.00)	−0.083^a^	0.934
Female	119 (41.18%)	38.00 (34.00, 42.00)		
BMI				
<18.5 kg/m^2^	33 (11.42%)	37.00 (31.50, 42.00)	12.752^b^	**0.002**
18.5 ~ 23.9 kg/m^2^	177 (61.25%)	39.00^(*)^ (36.00, 43.00)		
≥24 kg/m^2^	79 (27.34%)	36.00^(*)^ (33.00, 40.00)		
Ethnicity				
Han	270 (93.43%)	39.00 (35.00, 42.00)	−0.773^a^	0.440
Others	19 (6.57%)	38.00 (32.00, 41.00)		
Habitual residence				
Cities and towns	219 (75.78%)	39.00 (35.00, 42.00)	−1.121^a^	0.262
Village	70 (24.22%)	37.50 (34.00, 41.25)		
Employment status				
Employed	153 (52.94%)	38.00 (34.00, 43.00)	−0.020^a^	0.984
Unemployed	136 (47.06%)	39.00 (35.00, 41.00)		
Education level				
Junior high school and below	56 (19.38%)	37.00 (33.00, 41.75)	4.844^b^	0.184
Senior high school or technical secondary school	84 (29.07%)	38.00 (34.00, 41.75)		
Junior college or bachelor	139 (48.10%)	39.00 (35.00, 42.00)		
Master and above	10 (3.46%)	39.00 (35.75, 43.00)		
Marital status				
No	60 (20.76%)	37.00 (32.50, 42.00)	−1.135^a^	0.256
Yes	229 (79.24%)	39.00 (35.00, 42.00)		
Insurance status				
Medicaid	283 (97.92%)	39.00 (35.00, 42.00)	−1.330^a^	0.183
Self-pay	6 (2.08%)	35.50 (26.75, 40.25)		
Economic burden				
No	49 (16.96%)	39.00 (36.00, 45.00)	−2.546^a^	0.011
Yes	240 (83.04%)	38.00 (34.00, 41.00)		
Time after transplantation				
3–6 months	23 (7.96%)	41.00^(*)^ (37.00, 47.00)	18.351^b^	**<0.001**
7–12 months	20 (6.92%)	39.00 (33.50, 42.00)		
13–60 months	137 (47.40%)	39.00^(†)^ (35.00, 43.00)		
>60 months	109 (37.72%)	37.00^(*†)^ (33.00, 40.00)		
Donor type				
Deceased donor	261 (90.31%)	39.00 (35.00, 42.00)	−0.366^a^	0.714
Living donor	28 (9.69%)	37.50 (34.00, 42.75)		
Rejection				
No	230 (79.58%)	39.00 (35.00, 42.00)	−2.290^a^	0.022
Yes	59 (20.42%)	37.00 (32.00, 41.00)		
Infection				
No	208 (71.97%)	39.00 (35.00, 43.00)	−1.679^a^	0.093
Yes	81 (28.03%)	38.00 (34.00, 41.00)		
Number of hospitalizations				
0 times	95 (32.87%)	39.00 (36.00, 42.00)	5.127^b^	0.077
1–2 times	119 (41.18%)	39.00 (34.00, 41.00)		
>2 times	75 (25.95%)	37.00 (33.00, 41.00)		

^a^Denotes Mann–Whitney U test; ^b^ denotes Kruskal-Wallis test. The same superscripts (^*†^) denote significant differences between the two groups following multiple comparisons. *p* < 0.05 are considered significant and are highlighted. Bolded *p*-values indicate significant differences between groups in *post-hoc* tests.

### 3.3 Correlation analyses between variables in the HPM

Table 3 illustrates that the dietary behaviour of KTRs was positively associated with perceived behaviour benefits, self-efficacy, attitude, and social support (*r* = 0.420, 0.123, 0.407, and 0.345, respectively; *p* < 0.05) and negatively associated with depression and perceived barriers (*r* = −0.385 and −0.351, respectively; *p* < 0.01).

**Table 3:** Correlation analyses among variables in the HPM.

	Depression	Perceived behaviour benefits	Perceived behaviour barriers	Self-efficacy	Attitude	Social support	Dietary behaviour
Depression	1.000						
Perceived behaviour benefits	−0.158**	1.000					
Perceived behaviour barriers	0.256**	−0.130*	1.000				
Self-efficacy	−0.039	0.311**	0.041	1.000			
Attitude	−0.245**	0.627**	−0.125*	0.309**	1.000		
Social support	−0.207**	0.338**	−0.067	0.311**	0.333**	1.000	
Dietary behaviour	−0.385**	0.420**	−0.351**	0.123*	0.407**	0.345**	1.000

**Significance level at 0.01; *Significance level at 0.05.

### 3.4 Path analysis exploring the influencing factors of dietary behaviour in KTRs

**Figure 2:**
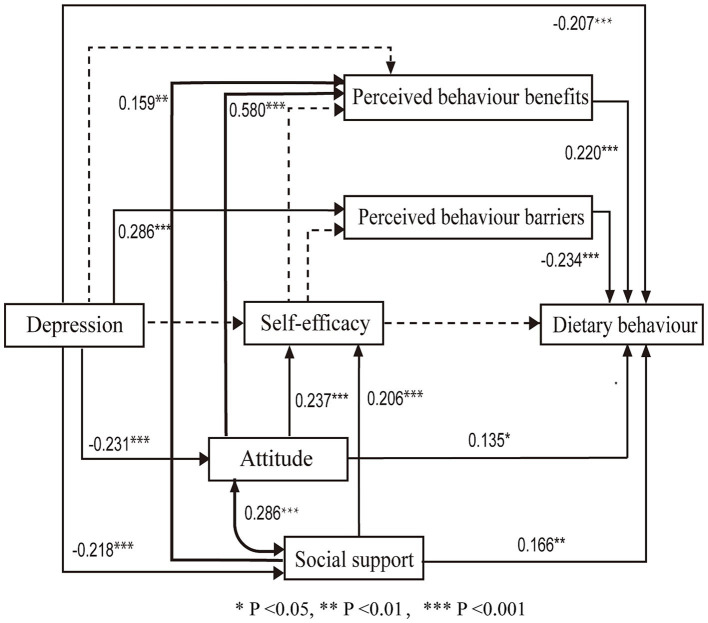
Standardized estimates in the path analysis. The associations among the variables are depicted with solid lines. The new lines are bold. The non-significant paths are depicted with dotted lines for comparison with the initial model. *P < 0.05, **P < 0.01, ***P < 0.001.

Non-parametric tests yielded statistically significant differences among different BMI levels, different financial burdens, post-transplantation time, and rejection situation, suggesting that these variables may be predictors of dietary behaviour. We used the above factors as control variables for dietary behaviour. Depression, attitude, perceived benefits, perceived barriers, social support, and self-efficacy, which were significantly correlated with dietary behaviour, entered into the path analysis model. But the initial hypothesized model exhibited inadequate fit, so direct paths from self-efficacy to dietary behaviour were removed, as well as the paths from depression to self-efficacy, the path from self-efficacy to perceived barriers, and the path from depression and self-efficacy to perceived benefits. Paths from attitude and social support to perceived benefits in KTRs and the connection between attitude and social support were incorporated (16, 25). The final path analysis results, illustrated in Figure 2, exhibited suitable fit indices: χ^2^/df = 2.079, RMSEA = 0.061, CFI = 0.945, TLI = 0.912, SRMR = 0.067. All correlations were statistically significant (*p* < 0.05).

Figure 2 summarises the crucial path associations. Table 4 displays the direct, indirect, and total effects on dietary behaviour. Depression (*β* = −0.207, *p* < 0.001), perceived behaviour benefits (*β* = 0.220, *p* < 0.001), perceived behaviour barriers (*β* = −0.234, *p* < 0.001), attitude (*β* = 0.135, *p* = 0.026) and social support (*β* = 0.166, *p* = 0.001) directly predicted dietary behaviour. Among these, depression, attitude, and social support exhibited indirect effects on dietary behaviour. The total effects of these five factors were 0.364, 0.220, −0.234, 0.935, and 0.545, respectively.

**Table 4:** Summary of direct and indirect effects of each factor on dietary behaviour.

Model path	Direct effect				Indirect effect	Total effect
	Estimate	S. E.	Z	*P-*value		
Depression→Dietary behaviour	−0.207	0.050	−4.148	***	–	0.364
Depression→Perceived behaviour barriers→Dietary behaviour	–	–	–	–	0.052	
Depression→Attitude→Dietary behaviour	–	–	–	–	−0.096	
Depression→Social support→Dietary behaviour	–	–	–	–	−0.115	
Depression→Attitude→Perceived behaviour benefits→Dietary behaviour	–	–	–	–	0.569	
Depression→Social support→Perceived behaviour benefits→Dietary behaviour	–	–	–	–	0.161	
Perceived behaviour benefits→Dietary behaviour	0.220	0.061	3.627	***	–	0.220
Perceived behaviour barriers→Dietary behaviour	−0.234	0.048	−4.861	***	–	−0.234
Attitude→Dietary behaviour	0.135	0.061	2.229	0.026	–	0.935
Attitude→Perceived behaviour benefits→Dietary behaviours	–	–	–	–	0.8	
Social support→Dietary behaviour	0.166	0.050	3.324	0.001	–	0.545
Social support→Perceived behaviour benefits→Dietary behaviour	–	–	–	–	0.379	

***p < 0.001.

In addition, depression predicted perceived barriers (*β* = 0.286, *p* < 0.001), attitude (*β* = −0.231, *p* < 0.001), and social support (*β* = −0.218, *p* < 0.001). Attitude (*β* = 0.580, *p* < 0.001; *β* = 0.237, *p* < 0.001) and social support (*β* = 0.159, *p* = 0.001; *β* = 0.206, *p* < 0.001) both directly predicted perceived benefits and self-efficacy. Notably, attitude and social support exhibited a positive correlation (*p* < 0.001). However, the VIF values for these two factors were both 1.115, indicating the absence of multicollinearity between them. Generally, all factors collectively accounted for 38.06% of the variance. Attitude, social support, depression, perceived barriers, and perceived benefits were important factors influencing dietary behaviour.

## 4 Discussion

This study, grounded in the HPM, investigated the current dietary behaviour and associated factors among KTRs. Several key findings were identified: (1) The dietary behaviour of KTRs was found to be at a moderate level. (2) Recipients with an absence of financial burdens, no history of rejection, normal BMI, and a shorter post-transplant duration exhibited better dietary behaviour. (3) Depression, perceived behaviour benefits, perceived behaviour barriers, attitude, and social support directly predicted dietary behaviour, while depression, attitude, and social support exhibited an indirect effect on dietary behaviour.

### 4.1 Dietary behaviour in kidney transplant recipients: a moderate level

In this study, KTRs had a dietary behaviour index score of 78.00%, indicating a moderate level. This finding is inconsistent with the finding of Lin’s study (26), which evaluated the adherence of KTRs to recommended dietary guidelines by measuring their daily nutrient intake compliance, revealing a notably low adherence rate. Such discrepancies may stem from recent advancements in information technology and the emergence of new health education methods. With the development of information technology, many newly developed applications such as MyFitnessPal can help KTRs calculate the amount of various nutrients in their diet, which makes their dietary compliance more convenient and feasible (27). Meanwhile, in recent years, kidney transplant physicians and follow-up professionals have paid more attention to health education and dietary risk monitoring in daily interventions, which may make the dietary behaviour scores of the participants in this study at a moderate level.

### 4.2 Influence of demographic and clinical characteristics on dietary behaviour

This study’s findings suggested that recipients with absence of financial burdens and no history of rejection exhibited better dietary behaviour. This observation can be attributed to the fact that recipients with a lower economic burden may find it easier to purchase low-fat and low-carbohydrate foods (28), which may contribute to improved dietary behaviour. Moreover, recipients without a history of rejection tend to achieve higher dietary behaviour scores, possibly because those with better dietary practices avoid foods that could affect drug concentrations, thereby reducing the risk of rejection (29). Additionally, our non-parametric analysis found that normal BMI and shorter post-transplant duration were associated with higher dietary behaviour scores. Subsequent multiple comparison analyses revealed that recipients with a normal BMI exhibited better dietary behaviour compared to those with higher BMI. This finding is consistent with previous research suggesting that recipients with a normal BMI are more inclined to consume a Mediterranean diet and exhibit better dietary behaviour (30). Furthermore, compared with the recipients whose postoperative period exceeded 60 months, those whose postoperative period was between 3 and 6 months or between 13 and 60 months performed better in terms of dietary behaviour scores. This trend suggests that as KTRs gradually return to normal life, increased social engagement and participation in daily activities may result in a reduced emphasis on dietary habits. Moreover, the effectiveness of dietary education for KTRs may decline as the follow-up period extends (31). These findings underscore the influence of demographic and clinical characteristics on the dietary behaviour of KTRs. Consequently, interventions aimed at improving dietary behaviour should be tailored to the specific circumstances of the recipients.

### 4.3 Direct and indirect effects of depression, and behaviour-specific cognitions and affect factors on dietary behaviour

This study identified both direct and indirect factors influencing dietary behaviour through path analysis. In terms of direct factors, the current study found that depression, perceived behaviour benefits, perceived behaviour barriers, attitude, and social support could directly predict dietary behaviour. A corpus of research on depression and dietary behaviour has illuminated a close association between patients’ psychological status and dietary behaviour (32). For instance, the adoption of the Mediterranean diet has been associated with a diminished incidence of depression (33), while a heightened risk of depression has been linked to escalated red meat consumption (34). This finding further demonstrated that an enhancement in perceived benefits could promote healthy dietary behaviour, consistent with previous findings (35). This may be attributed to the tendency of recipients who recognise the advantages of healthy behaviour to amend detrimental dietary habits. Conversely, research has shown that the greater individuals perceived dietary barriers, the more difficult it was for them to change their dietary behaviour and adhere to post-transplant dietary directives, ultimately resulting in lower dietary behaviour scores (36). Furthermore, it was found that recipients with positive attitudes are likely to exhibit better dietary habits. Research has indicated that patients with diabetics with a positive attitude exhibited a 3.2-fold likelihood (95% CI: 1.5–6.7) to follow dietary recommendations compared to their counterparts with negative attitudes (37). Additionally, we observed that social support positively influenced dietary behaviour in KTRs, corroborating the results of several previous studies (38, 39). This effect may be attributable to the proactive problem-solving strategies employed by recipients with high levels of social support, who are also more attentive to their post-operative health status, thereby enhancing their dietary behaviour (39). Future research should prioritise exploring interventions that target both direct pathways to enhance dietary behaviour.

This study also found that depression, attitude, and social support exhibited indirect effects on dietary behaviour. These results were different from those of previous studies, which primarily focused on direct associations (37) and did not consistently identify the same indirect pathways influencing behaviour (15, 40). In this study, depression exhibited an indirect association with dietary behaviour, which is broadly consistent with the theoretical underpinnings of the HPM (7). Depressed recipients may have a bad attitude and social support and thus perceive fewer benefits, which may lead to poor dietary behaviour. They may also have more perceived barriers, followed by poor dietary behaviour. Additionally, the current study indicated that attitude indirectly influenced dietary behaviour through perceived benefits, thus extending the pathways of attitude’s indirect effect on dietary behaviour. This finding is inconsistent with previous research, which has indicated that attitude, particularly instrumental attitude, may indirectly affect behaviour through factors such as behavioural intention (41, 42). Similarly, mirroring the indirect effect of attitude, social support also indirectly influenced dietary behaviour through perceived benefits, a finding that differs from prior studies (15). Previous research has suggested that social support may indirectly affect behaviour, such as fruit and vegetable intake, by influencing self-efficacy (15). These findings highlight the indirect effects of improving depression, fostering positive attitude, and bolstering social support to improve dietary behaviour. In this study, attitude and social support exhibited a positive correlation. A positive attitude enhances levels of food literacy (43). Furthermore, food literacy is most closely associated with social support among recipients post-transplant (44). Consequently, a favourable attitude may encourage recipients to actively seek social support. Additionally, beneficial social support factors, including social roles and communication patterns, significantly affect patients’ beliefs and attitudes (45). This further contributes to our understanding of how social support influences attitude.

### 4.4 Theoretical implications

This study extends the application of the HPM theory within the context of dietary behaviour among KTRs. In contrast to the initial hypothesised model, this study incorporated novel pathways from attitude and social support to perceived benefits in KTRs, as well as a connection between attitude and social support. However, some pathways from the initial model were also removed. In this study, there was no significant association between self-efficacy and depression, perceived benefits, or perceived barriers. Similarly, no significant correlation was observed between depression and perceived benefits. Overall, path analysis explained only 38.06% of the variance in dietary behaviour of KTRs, slightly lesser than the previous study (46). The slight reduction of explanatory power may result from the exclusion of commitment to action planning and immediate competing demands and preferences in our study. Therefore, in future research, the relationship between the variables in behaviour-specific cognition and affect warrants further examination.

### 4.5 Limitations

This study has several limitations. Firstly, the utilisation of self-reported questionnaires introduces the potential for reporting bias. Secondly, being a cross-sectional study, causal inferences are not supported. Thirdly, although our data were collected from four hospitals across different regions, the sample size is relatively small, which may result in selection bias and limit the generalisability of the findings to participants from diverse cultural backgrounds and regions. Future research should consider increasing the sample size and employing stratified or random sampling methods to reduce bias and better account for inter-hospital variability.

## 5 Conclusion

This study revealed that the scores of dietary behaviour among KTRs were at a moderate level and were notably influenced by psychological factors and behaviour-specific cognitive factors. Depression, perceived benefits, perceived barriers, attitude, and social support directly predicted dietary behaviour, and that depression, attitude, and social support exhibited indirect effects on dietary behaviour. These findings suggest that intervention about changing poor dietary attitude and negative mode, guiding them to receive more social support and perceived benefits, reducing their perceived barriers about dietary behaviour should be given. Healthcare professionals can also explore additional strategies, such as utilising digital behaviour change interventions, to enhance long-term dietary adherence in KTRs.

## Data Availability

The datasets presented in this article are not readily available because the dataset may be protected by copyright, and its use may require permission or licensing from the corresponding author. Requests to access the datasets should be directed to Hongxia Liu, hongxia_t@163.com.
